# Effect of eucalyptol on matrix metalloproteinase-9 and its tissue inhibitor in hypertensive rats

**DOI:** 10.6026/97320630019562

**Published:** 2023-05-31

**Authors:** Hussam Murad

**Affiliations:** 1 Department of Pharmacology, Faculty of Medicine, Rabigh campus, King Abdulaziz University, Jeddah, 21589, Saudi Arabia

**Keywords:** Eucalyptol, Lisinopril, Matrix metalloproteinase-9 (MMP-9), Tissue inhibitor of metalloproteinase (TIMP-1), Nicotine, Hypertension

## Abstract

Effects of eucalyptol, a key component of eucalyptus globules, on matrix metalloproteinase-9 (MMP-9) and its tissue inhibitor (TIMP-1), compared with lisinopril, were investigated in a model of hypertension induced by chronic intraperitoneal (IP) injection of low dose nicotine in rats. The hypertensive rats were randomly allocated to 4 groups (n=8): Positive control (PC, untreated), eucalyptol-treated group (1.0 mg/kg, IP), lisinopril-treated group (10 mg/kg, IP), and eucalyptol+lisinopril-treated group. Systolic blood pressure and plasma levels of MMP-9 and TIMP-1 were measured. All treatments decreased the elevated blood pressure and plasma levels of MMP-9 and TIMP-1 most significantly with the combination group which showed non-significant differences from the normal control group. Lisinopril reduced plasma levels of MMP-9 and TIMP-1 more significantly than eucalyptol. In conclusion, eucalyptol significantly decreased the increased plasma levels of MMP-9 and TIMP-1 in nicotine-induced hypertension in rats. Moreover, its combination with lisinopril exerted more significant effects compared to each drug alone. This makes this combination particularly useful in hypertension and related cardiovascular disorders where suppression of MMP-9 and TIMP-1 activities decreases the related complications and improves the overall morbidity and mortality. To our knowledge, the current data are novel, and may open the way for development of a co-delivery system of both drugs which could be beneficial in treatment of hypertension in chronic smokers.

## Background:

Matrix metalloproteinases (MMPs) play an essential role in the degradation of many constituents of the extracellular matrix in both normal conditions and pathological disorders. The expression and activities of MMP-2 and MMP-9 increase in many clinical cardiovascular conditions and also in animal models related to them [1]. Additionally, plasma levels of MMPs have been correlated with risk of the atherosclerotic events and could be used as biomarkers for cardiovascular risk even in individuals without clinical diseases [2]. Hypertension has many complications which can increase MMP-9 activity in plasma and tissues, and MMP-9 was detected to be involved in occurrence of the retinal complications of hypertension [3]. Nicotine has been reported to increase oxidative stress leading to hypertension and lipid peroxidation [4]. Eucalyptol (1,8-cineole), a key component of eucalyptus globules, exerted antioxidant effects in animals [5] and also reduced the Ca++ influx in cardiovascular muscles and decreased the aortic pressure [6]. Lisinopril, an angiotensin converting enzyme inhibitor, significantly inhibited MMP-9 activity after myocardial infarction in hamsters [7]. Therefore, it is of interest to evaluate effects of eucalyptol, compared to lisinopril, on systolic blood pressure and plasma levels of MMP-9 and TIMP-1 in a rat model of hypertension induced by chronic exposure to nicotine.

## Methods:

## Induction of hypertension and Experimental group:

Nicotine liquid, eucalyptol liquid, and lisinopril were purchased from Sigma Chemical Company (St. Louis, MO, USA). Lisinopril was dissolved in distilled water. The study was approved by the Research ethics committee, Faculty of Pharmacy and was carried out in accordance with the guidelines for use of laboratory animals. Hypertension was induced by chronic administration of low dose nicotine as previously described [8,9]. Briefly, male Sprague Dawley rats (250-300 g) were used and maintained at regular conditions under 12 h light/dark cycles for a week for acclimatization. The normal control (NC) group was given saline while other rats were given 0.8 mg/kg/day nicotine by intraperitoneal (IP) injection for 21 days, followed by 5 mg/kg nicotine IP on day 22 to enhance effects of the low-dose nicotine. The hypertensive rats were randomly allocated to 4 groups (n=8): Positive control (PC, untreated hypertensive group), eucalyptol-treated group (1.0 mg/kg, IP) [9], lisinopril-treated group (10 mg/kg, IP) [10], and eucalyptol + lisinopril-treated group. The systolic blood pressure (SBP) was measured in the conscious rats non-invasively by using the tail cuff method by the NIBP system (AD Instruments Inc., CO, USA). The SBP was measured each day for 22 days, and lastly 30 minutes following the IP administration of eucalyptol and lisinopril on day 22. Animals were sacrificed one hour after the last SBP measurement.

## Determination of plasma matrix metalloproteinase-9 (MMP-9) and tissue inhibitor of metalloproteinase-1 (TIMP-1):

Rats were anaesthetized with isoflurane, blood samples were taken by cardiac puncture, and plasma was separated and stored at -80°C. The levels of MMP-9 and TIMP-1 were measured using ELISA kits according to the manufacturer's instructions (MyBioSource, Inc., San Diego, USA).

## Statistical analysis:

Data were expressed as means ± SEM. The SPSS software (version 22, USA) was used. Comparisons between the different groups were made using one-way analysis of variance (ANOVA) followed by Tukey test for multiple comparisons. The difference was considered significant when P < 0.05.

## Results:

## Effects of eucalyptol and lisinopril on systolic blood pressure in hypertensive rats:

Chronic administration of low-dose nicotine followed by a booster high dose of nicotine elevated the SBP compared with the normal control group. All treatments significantly decreased the elevated BP, compared with the hypertensive control group. The combination group showed the most significant reduction with a non-significant difference from the NC group (Table 1).

**Figure 1:**
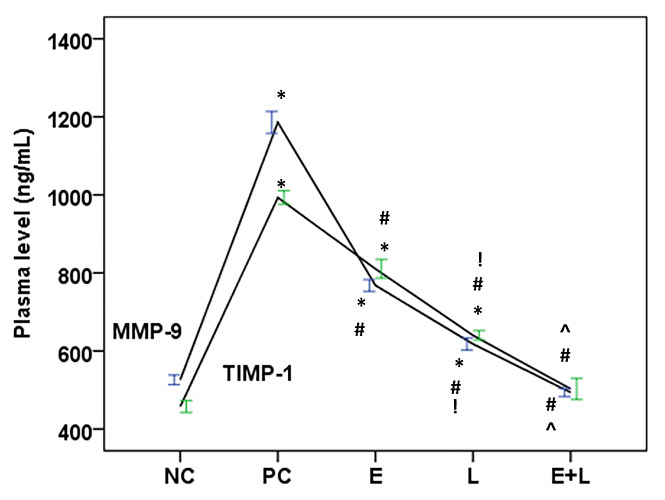
Effects of eucalyptol (E, 1.0 mg/kg, IP), lisinopril (L, 10 mg/kg, IP), and their combination (E+L) on plasma levels of MMP-9 and TIMP-1 in a model of intraperitoneal nicotine-induced hypertension in rats (n=8). Data are expressed as Mean ± SEM. For each biomarker: * P < 0.05 vs NC (normal control), # P < 0.05 vs PC (positive control), ! P < 0.05 vs eucalyptol, P < 0.05 vs eucalyptol and Lisinopril.

Effects of eucalyptol and lisinopril on plasma levels of MMP-9 and TIMP-1 in in hypertensive rats: The hypertensive rats showed significant increases of the plasma levels of MMP-9 and TIMP-1 compared to the NC group. All treatments significantly reversed these changes compared with the hypertensive control group. Lisinopril exerted more significant reductions than eucalyptol, while the combination group showed the most significant reductions with non-significant differences from the NC group (Figure 1).

## Discussion:

Hypertension is correlated with structural changes of the vascular extracellular matrix. MMPs and their inhibitors (TIMPs) play a key role in modification of this matrix. Disturbances of the balance between the MMPs and TIMPs are involved in the pathogenesis of cardiovascular disorders [11]. After myocardial infarction in hamsters, lisinopril significantly inhibited activities of both angiotensin converting enzyme and MMP-9 [7]. In a lipopolysaccharide-induced acute lung inflammation in BALB/C mice, intraperitoneal eucalyptol attenuated the inflammation-associated increases in MMP-9 expression indicating an anti-inflammatory effect. Thus, eucalyptol could be an important agent in the treatment of lung inflammation [12]. In rats chronically exposed to nicotine, 1,8-cineole significantly reduced SBP, increased plasma nitrite concentrations, and suppressed lipid peroxidation [9]. Nicotine was found to increase the expression and activities of the MMP-2 and MMP-9 [13]. Nicotine is involved in smoking-related cardiovascular ailments and may upregulate the MMP-2 and MMP-9. It was reported that nicotine induces the release of both MMP-2 and MMP-9 by the rat smooth muscle cells and that doxycycline (a non-selective MMP inhibitor) blocks the nicotine-induced vascular effects. Therefore, the nicotine-induced cardiovascular effects involve MMPs and hence MMPs inhibitors can counter these effects [14]. In a clinical trial, it was concluded that the increased plasma levels of MMP-9 and TIMP-1 at baseline in hypertensive patients could reflect an elevated deposition of type I collagen at the cost of other constituents in the cardiovascular ECM. Moreover, following management of the cardiovascular risk, MMP-9 levels decreased and TIMP-1 levels increased suggesting a potential role for these markers in hypertension [15].

## Conclusion:

Eucalyptol significantly decreased the increased plasma levels of MMP-9 and TIMP-1 in chronic nicotine-induced hypertension in rats and its combination with lisinopril exerted more significant effects compared to each drug alone. This combination could be useful in hypertension and related cardiovascular disorders because suppression of MMP-9 and TIMP-1 activities will decrease the complications and improves the overall morbidity and mortality. To our knowledge, the current data are novel, and may open the way for development of a co-delivery system of both drugs which could be beneficial in treatment of hypertension in chronic smokers.

## Limitations of the study:

The outcomes were measured at only one time-point. The doses were chosen based on previous literature and a pilot study, however there is no full evidence for dose-dependent effects.

## Funding:

This research work was funded by Institutional Fund Projects under grant no. (IFPIP:44-828-1443). The authors gratefully acknowledge technical and financial support provided by the Ministry of Education and King Abdulaziz University, DSR, Jeddah, Saudi Arabia.

## Figures and Tables

**Table 1:** Effects of intraperitoneally-injected eucalyptol (1.0 mg/kg), lisinopril (10 mg/kg), and their combination on the systolic blood pressure (SBP) in a model of intraperitoneal nicotine-induced hypertension in rats (n=8).

	**NC**	**PC**	**Eucalyptol**	**Lisinopril**	**Eucalyptol + Lisinopril**
Mean BP (mmHg)	104.63±3.17	176.50±3.40 *	131.63±3.37 *,#	124.13±5.51 *,#	100.75±2.27 #
Data are expressed as Mean ± SEM. * P < 0.05 vs NC (normal control), # P < 0.05 vs PC (positive control), P < 0.05 vs eucalyptol (P < 0.001) and Lisinopril (P = 0.001).
